# The correlation of neutrophil-percentage-to-albumin ratio with chronic kidney disease risk among the United States diabetic population

**DOI:** 10.1097/MD.0000000000044691

**Published:** 2025-09-19

**Authors:** Jiachen Fan, Xin Meng, Yanfang Lu, Huixia Cao

**Affiliations:** a People’s Hospital of Zhengzhou University, Zhengzhou, Henan, China; b Department of Oral and Maxillofacial Surgery, Henan Provincial People’s Hospital, Zhengzhou, Henan, China; c Henan Provincial Key Laboratory of Kidney Disease and Immunology, Henan Provincial Clinical Research Center for Kidney Disease, Henan International Joint Laboratory of Kidney Disease and Microenvironment, Henan Provincial People’s Hospital, Zhengzhou, Henan, China.

**Keywords:** chronic kidney disease, diabetes, NHANES, NPAR

## Abstract

The neutrophil-percentage-to-albumin ratio (NPAR) is a novel biomarker. However, the relationship between the NPAR and the development of chronic kidney disease (CKD) in the diabetic population is still unclear. The purpose of this study was to investigate the potential association between the NPAR and CKD in the diabetic population. This cross-sectional study included participants in the National Health and Nutrition Examination Survey from 2009 to 2018. Analyses were performed via weighted multifactor logistic regression, subgroup analyses and weighted restricted cubic spline curves. Age, sex, race, family poverty-to-income ratio, education, smoking history, alcohol consumption, hypertension, hyperuricemia, hyperlipidemia, cardiovascular disease, and anemia were included as covariates. In addition to CKD, the estimated glomerular filtration rate and urinary albumin/creatinine ratio were used as outcomes to fit the models. A total of 2436 participants were included in the study after some who provided insufficient information were excluded. Weighted logistic regression models revealed that the NPAR was positively correlated with the incidence of CKD after controlling for all other variables (odds ratio: 1.13, 95% confidence interval: 1.06–1.20, *P* < .05). When NPAR was transformed into a categorical variable on the basis of quartiles, only the Q4 was independently associated with CKD incidence (odds ratio: 2.23, 95% confidence interval: 1.33–3.71, *P* < .05). There was a nonlinear relationship between CKD and NPAR (*P* < .05). Subgroup analyses revealed strong interactions between NPAR and age, male and hypertension (*P* < .05). The NPAR was positively correlated with an increased risk of CKD in the diabetic population. These results suggest that the NPAR may have potential utility in personalized management.

## 1. Introduction

Chronic kidney disease (CKD) is a disease characterized by a progressive decline in kidney function and has become a serious threat to the lives of millions of people worldwide.^[1]^ Numerous factors may contribute to CKD, including metabolic syndrome, diabetes, hypertension, primary renal disease, and other systemic disorders.^[2]^ Nevertheless, CKD often develops insidiously, and few patients are aware of poor kidney function while the disease first starts. Although the estimated glomerular filtration rate (eGFR) is frequently used in clinical practice to screen for and diagnose CKD, its application is limited by its low sensitivity to this condition.^[3]^ Therefore, finding accessible and highly sensitive indicators is quite important and valuable for therapeutic work.

Inflammation plays an important role in the pathogenesis and development of CKD. Owing to the reduced filtration capacity of the kidney, various metabolic wastes and toxins cannot be excreted in a timely manner. Elevated concentrations of inflammatory substances, including tumor necrosis factor-α, interleukin-6, and C-reactive protein, cause immunological imbalance and disturbances in the internal environment.^[4,5]^ These inflammatory factors impair tubular reabsorption and glomerular filtration while harming podocytes, epithelial cells, and endothelial cells.^[6]^ Interestingly, some inflammatory organisms have some predictive value for CKD prognostic assessment and disease progression. For example, the systemic immunoinflammatory index, platelet–lymphocyte ratio and neutrophil–lymphocyte ratio are highly correlated with poor prognosis in patients with renal disease.^[7]^ These indicators can be widely employed at the grassroots level and are cost-effective because they are further computed via standard blood tests.

Several recent studies have shown that the neutrophil-percentage-to-albumin ratio (NPAR) can be used to predict a variety of diseases.^[8–11]^ The NPAR is calculated by dividing the percentage of neutrophil molecules by the serum level, reflecting the level of inflammation in the body.^[12]^ The NPAR has recently been linked to an elevated risk of developing CKD among the general population.^[13]^ It is concerning that hypoproteinemia is a common symptom of renal failure brought on by diabetes mellitus.^[14]^ More research is needed to determine whether decreased blood levels have an impact on the connection between the NPAR and CKD. In this study, we sought to examine the association between the NPAR and CKD in participants with diabetes of the National Health and Nutrition Examination Survey (NHANES).

## 2. Methods

### 2.1. Study design and population

All data for this study were obtained from the NHANES. The data collected by the NHANES are extremely broad and cover health information on different populations in the United States, including basic individual information, interview data, physical measurements, and laboratory test data. The large sample size, a complex, multistage, probability sampling design was used in the data collection process. To ensure that the results of the study truly reflect the health status of the U.S. population, each individual was assigned a certain weight to reduce the bias present in the sampling process. The survey was approved by the National Center for Health Statistics (NCHS) Ethics Review Committee. This research was performed in accordance with relevant guidelines from the NHANES and the Declaration of Helsinki. All participants provided written informed consent.

**Figure 1.**
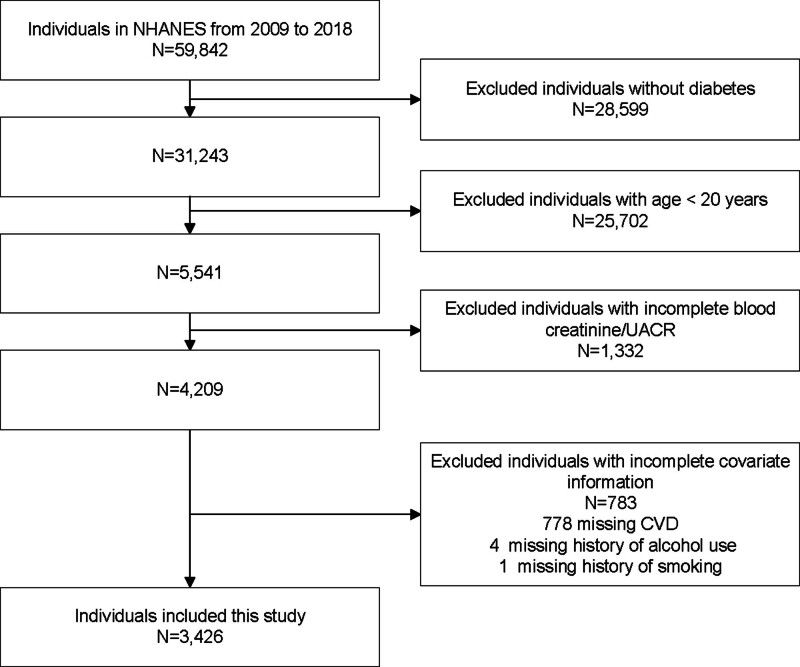
Flow chart for inclusion and exclusion of individuals. CVD = cardiovascular disease, NHANES = National Health and Nutrition Examination Survey, UACR = urinary albumin/creatinine ratio.

Our study included data from 6 survey cycles: 2009–2010, 2011–2012, 2013–2014, 2015–2016, and 2017–2018. Initially, 59,842 individuals were enrolled. A total of 3426 individuals were included in our study after individuals without diabetes (n = 28,599), individuals under the age of 20 (n = 25,702), individuals with incomplete blood creatinine/urinary albumin/creatinine ratio (UACR) (n = 1332), and individuals with incomplete covariate information (n = 783) (778 without cardiovascular disease [CVD], 4 without a history of alcohol use and 1 without a history of smoking) were excluded (Fig. 1).

### 2.2. Calculation of NPAR

The proportion of neutrophils in the blood was determined via the Coulter VCS system of the Coulter HMX Hematology Analyzer, as directed by the NHANES. The DcX800 method was used to measure the albumin concentration. Next, the NPAR value was calculated using the following formula: albumin (g/dL)/neutrophil (%).

### 2.3. Evaluation of CKD

We used the CKD-EPI Creatinine Equation to calculate the eGFR.^[15]^ The equation is eGFR=142×minScr/κ, 1α×maxScr/κ,1-1.209 × 0.9938Age×1.012 if female, where Scr is the serum creatinine (mg/dL), κ is 0.7 (females) or 0.9 (males), α is ‐0.241 (females) or ‐0.302 (males), min indicates the minimum of Scr/κ or 1, and max indicates the maximum of Scr/κ or 1.

According to the description of the NHANES, urinary albumin is measured by a solid-phase fluorescent immunoassay. Urinary creatinine is measured using enzymatic methods in the Roche/Hitachi Modular P Chemistry Analyzer. The ratio of urinary albumin to urinary creatinine was subsequently calculated. When the eGFR is <60 mL/min/1.73 m^2^ and/or the UACR is higher than or equal to 30 mg/g, CKD can be diagnosed.^[16]^

### 2.4. Evaluation of diabetes

Diabetes mellitus was defined as follows: self-reported previous diagnosis of DM, insulin, fasting glucose ≥ 7 or glycohemoglobin ≥ 6.5%.

### 2.5. Covariates

Covariates, that is, confounders, which may interfere with the relationship between the NPAR and CKD. In this study, several factors that may contribute to CKD were included. These factors are derived from the NHANES and are collected by medical professionals. All covariates were divided into 3 categories: basic information [age, sex (male and female), race (Mexican American, other Hispanic, non-Hispanic White, non-Hispanic Black and other race—including multiracial), family poverty-to-income ratio (PIR, <1 and ≥ 3) and education (less than high school, high school graduates, and above high school)], lifestyle habits (smoking history and alcohol consumption), and kidney-related diseases [hypertension, hyperuricemia, hyperlipidemia, CVD, and anemia]. Drinking consumption was divided into 2 categories: nondrinkers (those who drank fewer than 12 drinks in their lifetime) and drinkers (those who drank more than 12 drinks in their lifetime). Smoking status was categorized into never smokers and smokers, based on whether or not an individual had smoked a total of 100 cigarettes in his or her lifetime. Hypertension was diagnosed if an individual had a self-reported history of hypertension or if the average of 3 systolic blood pressure measurements was ≥ 140 mm Hg and/or the average diastolic blood pressure was ≥ 90 mm Hg. Individuals with a diagnosis of heart failure, coronary heart disease, angina pectoris, or heart attack were considered to have CVD. Anemia was identified when hemoglobin was < 130 g/L for men and < 120 g/L for women. Hyperuricemia is ascertained based on blood uric acid concentrations, usually defined as values exceeding 420 μmol/L in men and above 360 μmol/L in women.^[17]^ Hyperlipidemia was diagnosed when any of the following conditions were met: triglycerides ≥ 1.69 mmol/L, total cholesterol ≥ 5.17 mmol/L, low-density lipoprotein ≥ 3.36 mmol/L, high-density lipoprotein < 1.03 mmol/L in males or < 1.29 mmol/L in females, or the prescription of lipid-lowering drugs.^[18]^

Specific questionnaires, laboratory test methods and quality control standards are detailed on the NHANES website (https://www.cdc.gov/nchs/nhanes/).

### 2.6. Statistical analysis

In consideration of the arbitrary and sporadic nature of complex sampling design, the application of weights during data analysis was undertaken in accordance with the stipulated guidelines provided by the NHANES. For incomplete data (missing rate of no more than 10%), the multiple imputations method was utilized by the mice R package. For continuous variables (e.g., uric acid, total cholesterol, triglycerides, low-density lipoprotein, high-density lipoprotein, hemoglobin, and blood pressure), the “pmm” (predictive mean matching) method was used, while for categorical variables (e.g., smoking status, alcohol consumption, and education level), the “logreg’ (logistic regression) method was applied. Continuous variables are described as the means ± standard deviations, and categorical variables are expressed as numbers and weighted percentages. To compare the baseline data, continuous variables were subjected to weighted t tests, whereas categorical variables were analyzed via weighted chi-square tests. In addition, the relationship between the NPAR and CKD was investigated via weighted logistic regression. Three regression models were constructed to adjust for potential confounders. Model 1 was the original model, incorporating only 1 independent variable, the NPAR. Model 2 was adjusted for individuals” basic information and lifestyle factors (including age, sex, race, PIR, education level, smoking status, and alcohol consumption). The second model, diseases that may cause CKD or be associated with CKD (hypertension, hyperuricemia, hyperlipidemia, CVD, and anemia) were further included as covariates to construct Model 3. NPAR was stratified based on trichotomies (13.40 and 15.55), converted to categorical variables, and then refitted to the model. The weighted estimate of the receiver operating characteristic curve was plotted, and the area under the weighted estimate of the receiver operating characteristic curve (wAUC) values were calculated to evaluate the diagnostic performance of models.

Subgroup analysis was subsequently performed to assess the effect of NPAR on CKD in different populations. Age was transformed into a dichotomous variable based on whether it was younger than 60 years. The analysis was stratified according to various covariates. No adjustment for multiple comparisons was performed. All covariates in Model 3 were fitted except for the subgroup variables. *P* value for interaction was used to determine whether there were differences in the effects of the NPAR on CKD among the different subgroups. A weighted restricted cubic spline (RCS) was also constructed to explore the potential nonlinear relationship between the NPAR and CKD.

All statistical analyses were performed using R software, version 4.4.1. *P* < .05 was considered statistically significant.

## 3. Results

### 3.1. Characteristics of participants

After the data were processed in accordance with strict inclusion and exclusion criteria, 3426 individuals were included in our study. These individuals had a mean age of 57.67 ± 0.39 years; 53.59% were males. In our cohort, the weighted prevalence of CKD reached 7.96%, which is almost comparable to the global CKD prevalence.^[19]^ Participants were divided into 2 groups based on their CKD status. The mean NPAR level was higher among participants with CKD compared to those without CKD. Age, PIR, education, alcohol consumption, hypertension, hyperuricemia, CVD, anemia, and NPAR were significantly different between the CKD and non-CKD groups (*P* < .05). However, no significant differences were observed between 2 groups with respect to sex, ethnicity, smoking status, or hyperlipidemia. The detailed clinical data are recorded in Table 1.

**Table 1:** Baseline characteristics of participants.

Variables	CKD (n = 3106)	Non-CKD (n = 320)	*P* value
Sex, n (%)			.156
Male	1632 (54.1%)	173 (47.1%)	
Female	1474 (45.9%)	147 (52.9%)	
Age, mean ± SD	56.66 ± 14.33	69.46 ± 10.98	<.001
Race, n (%)			.097
Mexican American	617 (11.7%)	50 (7.7%)	
Other Hispanic	366 (6.2%)	26 (4.3%)	
Non-Hispanic White	975 (56.5%)	121 (60.3%)	
Non-Hispanic Black	815 (16.3%)	97 (19.3%)	
Other race (including multiracial)	333 (9.3%)	26 (8.4%)	
PIR, n (%)			.001
<1.0	790 (18.1%)	95 (25.5%)	
1.0–3.0	1409 (40.4%)	155 (46.5%)	
≥3.0	907 (41.6%)	70 (28.0%)	
Education, n (%)			.028
Below high school	583 (11.1%)	81 (17.8%)	
High school	536 (14.4%)	61 (16.3%)	
Above high school	1987 (74.5%)	178 (65.8%)	
Smoke, n (%)	1521 (50.6%)	173 (51.0%)	.897
Alcohol consumption, n (%)	1992 (68.6%)	190 (56.3%)	<.001
Hyperuricemia, n (%)	718 (23.0%)	177 (55.9%)	<.001
Hypertension, n (%)	2114 (66.0%)	294 (91.8%)	<.001
Anemia, n (%)	461 (12.2%)	142 (42.4%)	<.001
CVD, n (%)	538 (17.4%)	118 (33.3%)	<.001
Hyperlipidemia, n (%)	2823 (92.0%)	297 (92.7%)	.633
NPAR, mean ± SD	14.35 ± 2.74	15.96 ± 3.27	<.001

CKD = chronic kidney disease, CVD = cardiovascular disease, NPAR = neutrophil-percentage-to-albumin ratio, PIR = poverty income ratio, SD = standard derivation.

### 3.2. Association of the NPAR with CKD

Next, we fitted weighted logistic regression models to assess the association between NPAR and CKD. Our study indicated that the NPAR was positively associated with CKD (odds ratio [OR] = 1.21, 95% confidence interval [CI] = 1.14–1.29) (Table 2). After adjusting for multiple covariates, NPAR remained an independent risk factor for CKD (Model 2: OR = 1.18, 95% CI = 1.11–1.26; Model 3: OR = 1.13; 95% CI = 1.06–1.20).

**Table 2:** Association between the NPAR and CKD.

Exposure	Model 1		Model 2		Model 3	
	OR (95% CI)	*P* value	OR (95% CI)	*P* value	OR (95% CI)	*P* value
NPAR (continuous)	1.21 (1.14, 1.29)	<.001	1.18 (1.11, 1.26)	<.001	1.13 (1.06, 1.20)	<.001
NPAR (categories)						
Quartile 1	Reference		Reference		Reference	
Quartile 2	1.02 (0.62, 1.67)	.941	0.99 (0.60, 1.62)	.590	0.98 (0.56, 1.72)	.953
Quartile 3	1.35 (0.82, 2.22)	.239	1.24 (0.75, 2.07)	<.398	1.15 (0.66, 2.00)	.622
Quartile 4	3.46 (2.20, 5.43)	<.001	2.94 (1.82, 4.77)	<.001	2.23 (1.33, 3.71)	.003

Model 1: crude model; Model 2: adjusted for age, sex, race, PIR, education level, smoking status, and alcohol consumption; Model 3: adjusted for age, sex, race, PIR, education level, smoking status, alcohol consumption hypertension, hyperuricemia, hyperlipidemia, cardiovascular disease, and anemia.

CKD = chronic kidney disease, NPAR = neutrophil-percentage-to-albumin ratio, OR= odds ratio.

After the NPAR was transformed into a categorical variable, we again fitted the model and observed a similar phenomenon. Notably, the associations between NPAR and CKD were not statistically significant across all categories. Only the Q4 group demonstrated a significant correlation between NPAR and CKD (Model 1: OR = 3.46, 95% CI = 2.20–5.43; Model 2: OR = 2.94, 95% CI = 1.82–4.77; Model 3: OR = 2.23, 95% CI = 1.33–3.71). The risk of CKD was elevated 2.23-fold for each unit increase in NPAR compared with that in Q1.

**Figure 2.**
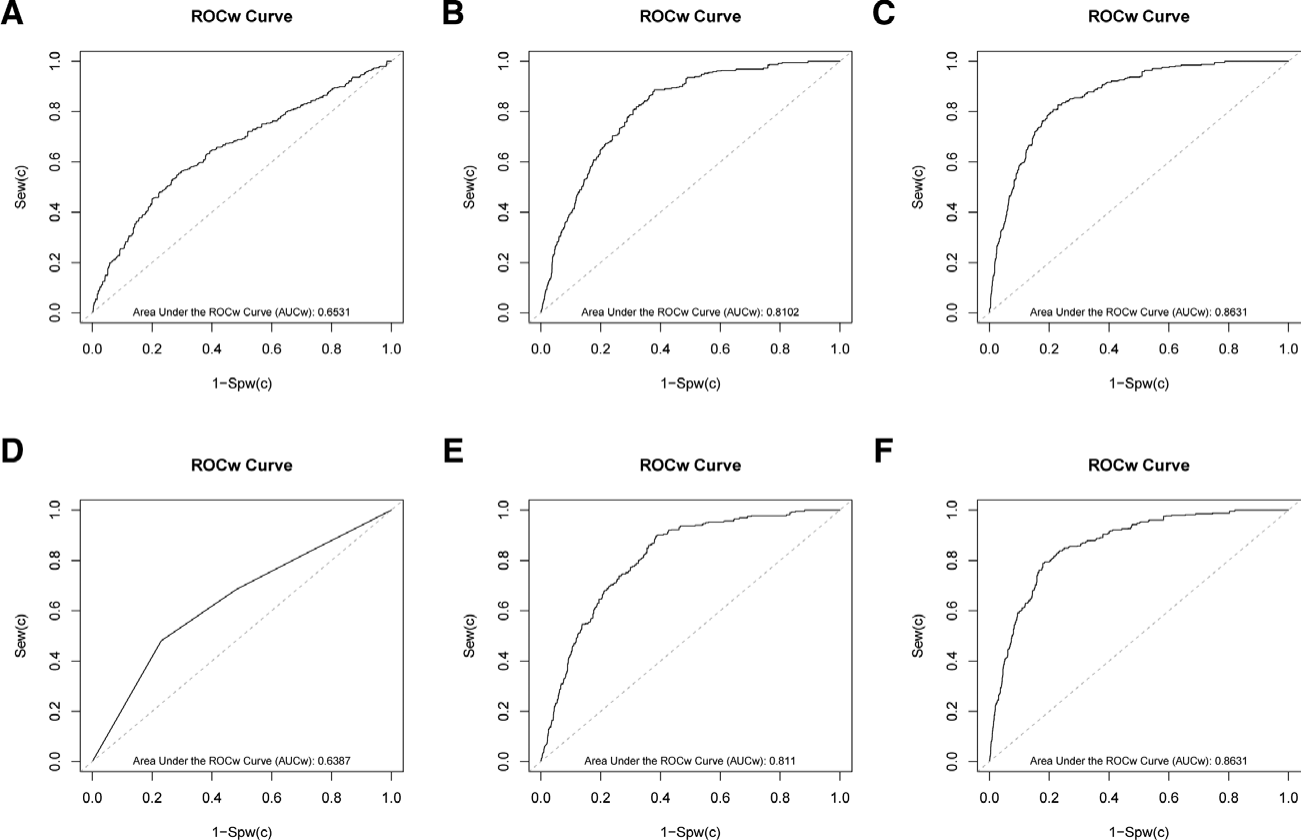
Weighted receiver operating characteristic curves for the 3 models. Sew = the weighted estimate of the sensitivity, Spw = the weighted estimate of the specificity, wAUC = the weighted estimate of the area under the ROC curve, wROC = the weighted estimate of the receiver operating characteristic curve.

To evaluate the prediction of models, we employed the weighted receiver operating characteristic curve analysis using the R package svyROC (Fig. 2). The wAUCs for the 3 models were 0.6531, 0.8103, and 0.8631, respectively. Although Model 1 demonstrated modest discriminatory ability, the wAUCs increased substantially after covariate adjustment. The categorical NPAR models showed a comparable diagnostic capability (Table 3).

**Table 3:** wAUCs for the models.

Exposure	Model 1	Model 2	Model 3
NPAR (continuous)	0.6531	0.8102	0.8631
NPAR (categories)	0.6387	0.8110	0.8631

Model 1: crude model; Model 2: adjusted for age, sex, race, PIR, education level, smoking status, and alcohol consumption; Model 3: adjusted for age, sex, race, PIR, education level, smoking status, alcohol consumption hypertension, hyperuricemia, hyperlipidemia, cardiovascular disease, and anemia.

NPAR = neutrophil-percentage-to-albumin ratio, wAUC = the weighted estimate of the area under the ROC curve.

### 3.3. Associations between NPAR and kidney biomarkers

According to clinical guidelines, the diagnosis of CKD depends on the levels of eGFR and UACR. Therefore, we fitted additional models with eGFR and UACR as dependent variables to verify the robustness of our findings. Individuals with eGFR below 60 mL/min/1.73 m² were classified as the low eGFR group. Consistent with the primary CKD model, higher NPAR levels were positively associated with low eGFR after full covariate adjustment (Model 3: OR = 1.12, 95% CI = 1.07–1.18), and participants in the highest NPAR quartile (Q4) had a 151% higher risk compared with those in the lowest quartile (Q1) (Model 3: OR = 2.51, 95% CI = 1.68–3.74) (Table S1, Supplemental Digital Content, https://links.lww.com/MD/Q60).

Furthermore, when participants were stratified by the presence of proteinuria (UACR ≥ 30 mg/g), higher NPAR remained significantly associated with increased risk. In fully adjusted models, participants in Q3 and Q4 had a 91% higher risk of proteinuria compared with those in Q1 (Model 3: OR = 1.91, 95% CI = 1.45–2.52) (Table S2, Supplemental Digital Content, https://links.lww.com/MD/Q60).

When eGFR and UACR were analyzed as continuous variables using weighted linear regression, results were consistent with those from weighted logistic regression (Tables S3 and S4, Supplemental Digital Content, https://links.lww.com/MD/Q60). These additional analyses corroborate the consistent association of higher NPAR with impaired renal function, further supporting the robustness of our findings.

### 3.4. Subgroup analysis

**Figure 3.**
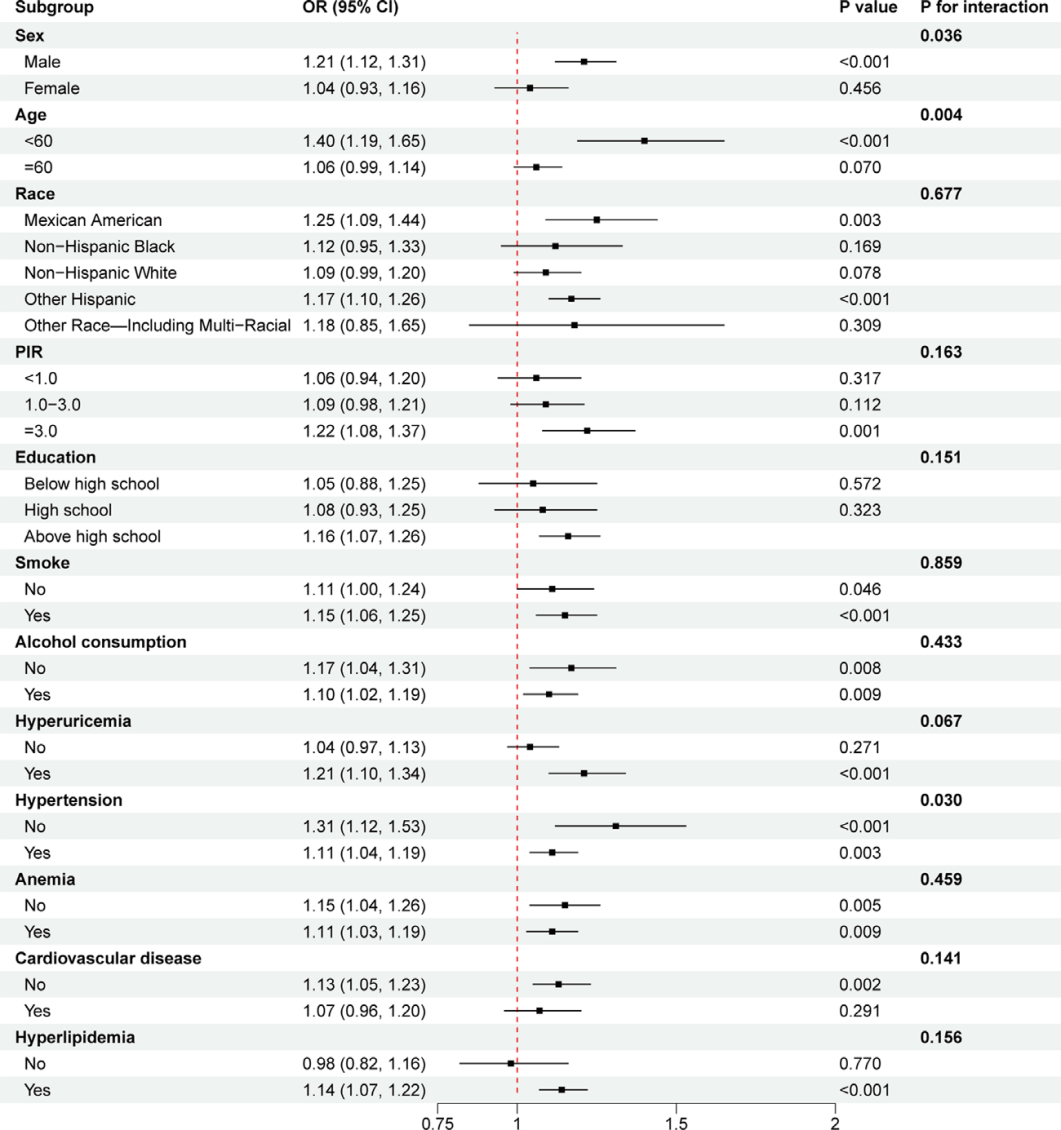
Associations between NPAR and CKD in different subgroups. All covariates were included in the models except for the subgroup variable of interest. Stronger associations were observed among younger participants, males, and individuals with hypertension. CKD = chronic kidney disease, NPAR = neutrophil-percentage-to-albumin ratio, OR = odds ratio; 95% CI = 95% confidence interval.

Subgroup analyses were conducted to ascertain the potential relationships between NPAR and CKD in different subgroups. NPAR tended to promote the development of CKD in almost all subgroups of the population, except for all those without hyperlipidemia (Fig. 3). However, only sex, age and hypertension significantly influenced the effect of the NPAR on CKD (*P* for interaction < .05). No significant interaction was observed when race, PIR, education level, smoking status, alcohol consumption, hyperuricemia, anemia, CVD and hyperlipidemia were used as stratification variables.

### 3.5. RCS regression

**Figure 4.**
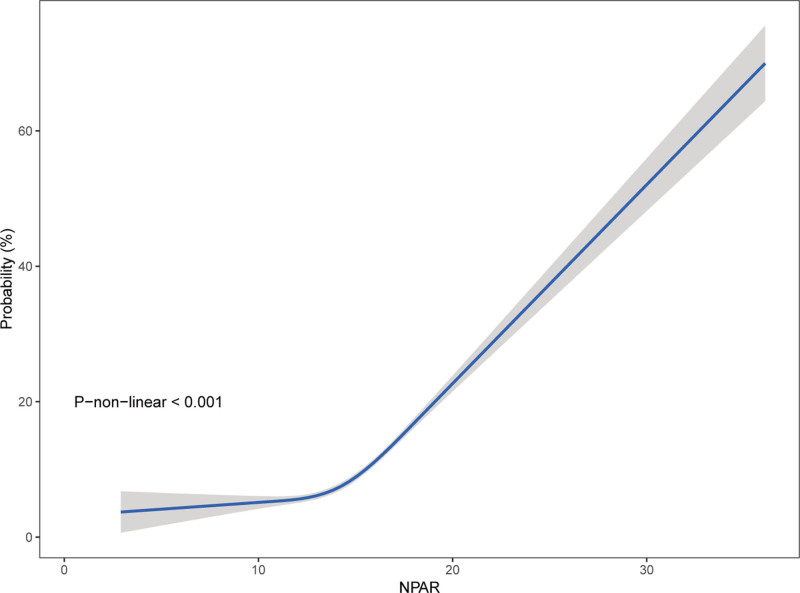
Nonlinear association between NPAR and CKD estimated by weighted restricted cubic spline regression. Although statistical nonlinearity was detected, the spline curve exhibited an approximately linear trend. The blue line represents the smooth curve fit between the variables, while the shaded gray area indicates the 95% confidence interval of the fit. CKD = chronic kidney disease, NPAR = neutrophil-percentage-to-albumin ratio.

We constructed a weighted RCS regression to explore the nonlinear relationship between NPAR and CKD (Fig. 4). The RCS regression indicated a nonlinear association between the NPAR and CKD after adjusting for all covariates (*P* for nonlinear < .05), although the graphical representation revealed a more linear correlation.

## 4. Discussion

This national survey analyzed from 6 NHANES cycles (2009–2018) and identified NPAR as an independent risk factor for CKD in diabetic individuals. Restricted cubic spline analysis revealed a nonlinear relationship between NPAR and CKD. Notably, stronger associations were observed in hypertensive, male, and younger (≤60 years) subgroups. As a convenient and noninvasive marker, NPAR may serve as an effective predictor of CKD in diabetic patients.

Weighted logistic regression showed a significant association between continuous NPAR levels and CKD incidence, while categorical analysis demonstrated that only the highest quartile (Q4) was independently associated with CKD, consistent with the nonlinear pattern observed.

Previous studies have linked elevated NPAR with increased risk and mortality in cardiovascular diseases, diabetic retinopathy, and hemodialysis patients.^[20–22]^ Although previous studies have confirmed that there is a close correlation between CKD and NPAR, diabetic nephropathy is often accompanied by hypoalbuminemia, which may affect NPAR calculations.^[13,23]^ Therefore, we focused on the diabetic population to improve specificity.

The NPAR is calculated as the neutrophil percentage divided by albumin, which represents the systemic inflammation level. Neutrophils contribute to kidney injury via reactive oxygen species and neutrophil extracellular traps, including glomerular injury, interstitial fibrosis, chronic inflammation, and endothelial vascular dysfunction.^[24–26]^ Hypoalbuminemia is not only associated with reduced eGFR but also with adverse outcomes in CKD patients.^[27,28]^ It may be related to malnutrition, inflammation.^[29]^

Renal function declines with age.^[30]^ Hypertension people in our study cohort had a greater risk of CKD, which is consistent with previous findings.^[31]^ Diabetes-induced hypoxia of the small renal arteries is worsened by hypertension, which might result in chronic inflammation.^[32]^ When the data was stratified by sex, we discovered that CKD was more common in male patients with high NPAR levels. Sex differences observed here may relate to hormonal influences.^[33]^ Taken together, these mechanisms may help explain the association between NPAR and the risk of CKD observed in our study.

Our study has several advantages. We included a large sample size across multiple survey cycles and ethnicities, which reduces bias from unbalanced sampling. Additionally, adjusting for various conditions related to CKD enhanced the robustness of our findings.

However, several limitations should be noted. First, our study population is limited to the United States, which may limit the generalizability of the results to other populations globally. Second, as a cross-sectional study, it cannot establish causality between NPAR and CKD; longitudinal studies are needed to clarify temporal relationships. Third, although NPAR is a convenient and noninvasive biomarker reflecting systemic inflammation, it may be influenced by other factors such as acute infections or comorbidities. Finally, our findings have not yet been validated in external cohorts, and future studies should aim to confirm these associations in different populations.

## 5. Conclusions

In this study, we found a positive correlation between NPAR levels and CKD. These findings suggest that high NPAR levels may be associated with the development of CKD in individuals with diabetes. However, more research is required to ascertain the mechanisms between NPAR and CKD, particularly in those with diabetes.

## Author contributions

**Conceptualization:** Jiachen Fan, Xin Meng, Huixia Cao.

**Data curation:** Xin Meng.

**Formal analysis:** Jiachen Fan, Xin Meng.

**Funding acquisition:** Huixia Cao.

**Investigation:** Jiachen Fan.

**Methodology:** Jiachen Fan, Xin Meng.

**Project administration:** Yanfang Lu, Huixia Cao.

**Resources:** Huixia Cao.

**Software:** Jiachen Fan.

**Supervision:** Yanfang Lu, Huixia Cao.

**Validation:** Jiachen Fan, Xin Meng.

**Visualization:** Jiachen Fan.

**Writing – original draft:** Jiachen Fan.

**Writing – review & editing:** Xin Meng, Yanfang Lu, Huixia Cao.

## Supplementary Material


