# Supplementary material for: The correlation of neutrophil-percentage-to-albumin ratio with chronic kidney disease risk among the United States diabetic population
Source: Medicine (Baltimore). 2025 Sep 19;104(38):e44691. doi: 10.1097/MD.0000000000044691 (PMC12459595; doi:10.1097/MD.0000000000044691)
Supplement: Supplementary file 1 [file medi-104-e44691-s001.docx]

Supplement Table 1. Association between the NPAR and the low eGFR group

| Exposure | Model 1 | | Model 2 | | Model 3 | |
| --- | --- | --- | --- | --- | --- | --- |
|  | OR (95% CI) | P value |  |  |  |  |
| NPAR | 1.18 (1.13, 1.23) | <0.001 | 1.16 (1.11, 1.21) | <0.001 | 1.12 (1.07, 1.18) | <0.001 |
| NPAR categories |  |  |  |  |  |  |
| Quartile 1 | Reference |  | Reference |  | Reference |  |
| Quartile 2 | 1.18 (0.79, 1.76) | 0.941 | 1.17 (0.75, 1.82) | 0.490 | 1.19 (0.72, 1.97) | 0.491 |
| Quartile 3 | 1.35 (0.79, 1.79) | 0.239 | 1.08 (0.68, 1.71) | 0.746 | 0.97 (0.58, 1.61) | 0.891 |
| Quartile 4 | 3.29 (2.39, 4.52) | <0.001 | 3.01 (2.15, 4.22) | <0.001 | 2.51 (1.68, 3.74) | <0.001 |

Weighted logistic regression model. Model 1: crude model; Model 2: adjusted for age, sex, race, PIR, education level, smoking status and alcohol consumption; Model 3: adjusted for age, sex, race, PIR, education level, smoking status, alcohol consumption hypertension, hyperuricemia, hyperlipidemia, cardiovascular disease and anemia. NPAR, neutrophil percentage-to-albumin ratio; eGFR, estimated glomerular filtration rate; OR, odds ratio.

Supplement Table 2. Association between the NPAR and the proteinuria group

| Exposure | Model 1 | | Model 2 | | Model 3 | |
| --- | --- | --- | --- | --- | --- | --- |
|  | OR (95% CI) | P value |  |  |  |  |
| NPAR | 1.18 (1.14, 1.23) | <0.001 | 1.17 (1.13, 1.23) | <0.001 | 1.15 (1.10, 1.20) | <0.001 |
| NPAR categories |  |  |  |  |  |  |
| Quartile 1 | Reference |  | Reference |  | Reference |  |
| Quartile 2 | 1.14 (0.88, 1.48) | 0.322 | 1.10 (0.85, 1.43) | 0.468 | 1.08 (0.84, 1.39) | 0.541 |
| Quartile 3 | 1.86 (1.46, 2.37) | <0.001 | 1.84 (1.43, 2.35) | <0.001 | 1.74 (1.37, 2.22) | <0.001 |
| Quartile 4 | 2.82 (2.10, 3.80) | <0.001 | 2.67 (1.99, 3.58) | <0.001 | 2.28 (1.71, 3.04) | <0.001 |

Weighted logistic regression model. Model 1: crude model; Model 2: adjusted for age, sex, race, PIR, education level, smoking status and alcohol consumption; Model 3: adjusted for age, sex, race, PIR, education level, smoking status, alcohol consumption hypertension, hyperuricemia, hyperlipidemia, cardiovascular disease and anemia. NPAR, neutrophil percentage-to-albumin ratio; OR, odds ratio.

Supplement Table 3. Association between the NPAR and eGFR

| Exposure | Model 1 | | Model 2 | | Model 3 | |
| --- | --- | --- | --- | --- | --- | --- |
|  | OR (95% CI) | P value | OR (95% CI) | P value | OR (95% CI) | P value |
| NPAR | 0.96 (0.95, 0.97) | <0.001 | 0.98 (0.96, 0.98) | <0.001 | 0.98 (0.97, 0.99) | <0.001 |
| NPAR categories |  |  |  |  |  |  |
| Quartile 1 | Reference |  | Reference |  | Reference |  |
| Quartile 2 | 0.97 (0.92, 1.02) | 0.213 | 1.00 (0.96, 1.05) | 0.904 | 1.00 (0.95, 1.05) | 0.956 |
| Quartile 3 | 0.93 (0.88, 0.99) | 0.018 | 0.97 (0.92, 1.03) | 0.350 | 0.99 (0.93, 1.04) | 0.616 |
| Quartile 4 | 0.75 (0.69, 0.82) | <0.001 | 0.82 (0.76, 0.88) | <0.001 | 0.86 (0.80, 0.92) | <0.001 |

Weighted linear regression model. Model 1: crude model; Model 2: adjusted for age, sex, race, PIR, education level, smoking status and alcohol consumption; Model 3: adjusted for age, sex, race, PIR, education level, smoking status, alcohol consumption hypertension, hyperuricemia, hyperlipidemia, cardiovascular disease and anemia. NPAR, neutrophil percentage-to-albumin ratio; eGFR, estimated glomerular filtration rate; OR, odds ratio.

Supplement Table 4. Association between the NPAR and UACR

| Exposure | Model 1 | | Model 2 | | Model 3 | |
| --- | --- | --- | --- | --- | --- | --- |
|  | OR (95% CI) | P value | OR (95% CI) | P value | OR (95% CI) | P value |
| NPAR | 1.13 (1.09, 1.18) | <0.001 | 1.12 (1.08, 1.17) | <0.001 | 1.10 (1.06, 1.15) | <0.001 |
| NPAR categories |  |  |  |  |  |  |
| Quartile 1 | Reference |  | Reference |  | Reference |  |
| Quartile 2 | 1.15 (0.85, 1.54) | 0.362 | 1.12 (0.83, 1.51) | 0.443 | 1.10 (0.81, 1.49) | 0.530 |
| Quartile 3 | 1.91 (1.45, 2.52) | <0.001 | 1.89 (1.42, 2.53) | <0.001 | 1.83 (1.36, 2.46) | <0.001 |
| Quartile 4 | 2.27 (1.78, 2.92) | <0.001 | 2.16 (1.66, 2.81) | <0.001 | 1.91 (1.45, 2.52) | <0.001 |

Weighted linear regression model. Model 1: crude model; Model 2: adjusted for age, sex, race, PIR, education level, smoking status and alcohol consumption; Model 3: adjusted for age, sex, race, PIR, education level, smoking status, alcohol consumption hypertension, hyperuricemia, hyperlipidemia, cardiovascular disease and anemia. NPAR, neutrophil percentage-to-albumin ratio; OR, odds ratio.
